# Multifunctional transcription factor TFII-I is an activator of BRCA1 function

**DOI:** 10.1038/bjc.2011.75

**Published:** 2011-03-15

**Authors:** M Tanikawa, O Wada-Hiraike, S Nakagawa, A Shirane, H Hiraike, S Koyama, Y Miyamoto, K Sone, T Tsuruga, K Nagasaka, Y Matsumoto, Y Ikeda, K Shoji, K Oda, H Fukuhara, K Nakagawa, S Kato, T Yano, Y Taketani

**Affiliations:** 1 Department of Obstetrics and Gynecology, Graduate School of Medicine, The University of Tokyo, 7-3-1 Hongo, Bunkyo-ku, Tokyo 113-8655, Japan; 2 Department of Urology, Graduate School of Medicine, The University of Tokyo, 7-3-1 Hongo, Bunkyo-ku, Tokyo 113-8655, Japan; 3 Department of Radiology, Graduate School of Medicine, The University of Tokyo, 7-3-1 Hongo, Bunkyo-ku, Tokyo 113-8655, Japan; 4 Institute of Molecular and Cellular Biosciences, The University of Tokyo, 1-1-1 Yayoi, Bunkyo-ku, Tokyo 113-0034, Japan

**Keywords:** TFII-I, BRCA1, interaction, activation, DNA damage repair

## Abstract

**Background:**

The TFII-I is a multifunctional transcriptional factor known to bind specifically to several DNA sequence elements and to mediate growth factor signalling. A microdeletion at the chromosomal location 7q11.23 encoding TFII-I and the related family of transcription factors may result in the onset of Williams–Beuren syndrome, an autosomal dominant genetic disorder characterised by a unique cognitive profile, diabetes, hypertension, anxiety, and craniofacial defects. Hereditary breast and ovarian cancer susceptibility gene product BRCA1 has been shown to serve as a positive regulator of SIRT1 expression by binding to the promoter region of SIRT1, but cross talk between BRCA1 and TFII-I has not been investigated to date.

**Methods:**

A physical interaction between TFII-I and BRCA1 was explored. To determine pathophysiological function of TFII-I, its role as a transcriptional cofactor for BRCA1 was investigated.

**Results:**

We found a physical interaction between the carboxyl terminus of TFII-I and the carboxyl terminus of BRCA1, also known as the BRCT domain. Endogenous TFII-I and BRCA1 form a complex in nuclei of intact cells and formation of irradiation-induced nuclear foci was observed. We also showed that the expression of TFII-I stimulates the transcriptional activation function of BRCT by a transient expression assay. The expression of TFII-I also enhanced the transcriptional activation of the SIRT1 promoter mediated by full-length BRCA1.

**Conclusion:**

These results revealed the intrinsic mechanism that TFII-I may modulate the cellular functions of BRCA1, and provide important implications to understand the development of breast cancer.

The TFII-I was identified originally as a factor that could bind to two distinct promoter elements, the pyrimidine-rich initiator and the recognition site (E-box) for upstream factor 1. The TFII-I stimulates transcription from the potent TATA- and initiator-containing adenovirus major late promoter (AdMLP) synergistically with upstream factor 1 (Roy *et al*, 1997). The TFII-I is a unique multifunctional factor that selectively regulates gene expressions when activated by a variety of extracellular signals and can function both as a basal transcriptional factor and as an activator (Roy, 2007). An autosomal dominant genetic disorder Williams–Beuren syndrome is a multisystem disorder characterised by distinctive facial features, mental disability, diabetes mellitus, and supravalvular aortic stenosis. The haploinsufficiency for TFII-I is causative to the craniofacial phenotype in humans (Pober, 2010). The primary structure of TFII-I is compatible with its multifunctional properties, consisting of six direct reiterated I-repeats, R1–R6, each containing a putative helix-loop-helix motif (Roy, 2007). The I-repeats are postulated to be protein interaction surfaces. Given those multifunctional features and structural characteristics, it is important to investigate the role of the I-repeats in mediating protein interactions and to search for proteins that make complex with TFII-I through this domain.

The breast cancer susceptibility gene *BRCA1* encodes a phosphoprotein that is involved in ubiquitination, DNA damage response, regulation of cell cycle checkpoints, and transcriptional regulation. Binding to the transcriptional machinery by the carboxyl terminal BRCA1, referred to as BRCT domain, was the first biochemical activity ascribed to the BRCA1 protein (Anderson *et al*, 1998). In addition, BRCT has been shown to be involved in double-stranded DNA repair and homologous recombination (Callebaut and Mornon, 1997; Moynahan *et al*, 1999; Zhong *et al*, 1999). On the basis of the data that targeted deletion of the BRCT domain results in embryonic lethality (Hohenstein *et al*, 2001), BRCT is postulated to be indispensable for the normal cellular growth, and it would be intriguing to investigate physiological functions of BRCT. BRCA1 carboxyl-terminal domain (BRCT) possesses the autonomous transcriptional activation function when the BRCT domain is fused to a GAL4 DNA-binding domain (Monteiro *et al*, 1996). The point mutations in the BRCT domain derived from patients with inherited breast cancer result in loss of transcriptional activity (Chapman and Verma, 1996). The BRCT domain has been shown to be an interaction surface with a number of transcription factors and co-regulators (Saka *et al*, 1997; Wada *et al*, 2004; Oishi *et al*, 2006; Hiraike *et al*, 2010).

To better understand the functional significance and the transcriptional regulation of BRCT, we previously have searched for putative transcription coactivator complexes that interact with the BRCT domain using a biochemical approach (Wada *et al*, 2004; Oishi *et al*, 2006). During these studies, we preliminary found that TFII-I interacted with the BRCT domain. Here, we confirmed that the carboxyl terminus of TFII-I and BRCA1 form a complex *in vivo*. We further studied the effect on transcriptional regulation of BRCA1 driven by TFII-I. These findings establish a principal biological function of TFII-I as an activator of BRCA1 function, and identify TFII-I as a possible determinant of breast cancer.

## Materials and methods

### Cell culture

Human cervical adenocarcinoma HeLa (CCL-2), HCC1937 (CRL-2336) breast cancer cells that express a carboxyl-terminally truncated BRCA1 protein (Scully *et al*, 1999), and African green monkey kidney fibroblast-like COS7 (CRL-1651) cell lines were purchased from the American Type Culture Collection (Manassas, VA, USA). HeLa and COS7 cells were maintained in Dulbecco's modified Eagle medium supplemented with 10% fetal bovine serum. HCC1937 cells were maintained in RPMI medium supplemented with 10% fetal bovine serum.

### Plasmid construction

BRCA1 expression vectors, BRCT vectors and reporter constructs (17M8-AdMLP-luc and SIRT1-luc) were described previously (Wada *et al*, 2004; Hiraike *et al*, 2010). TFII-I expression vectors were described previously (Cheriyath and Roy, 2001).

### Chemicals and antibodies

Rabbit antibodies were anti-TFII-I, anti-BRCA1, and anti-GST (Cell Signaling Technology Inc., Temecula, CA, USA, catalogue no. #4562, #9010, and #2622, respectively). Mouse monoclonal antibodies were anti-BRCA1 (Calbiochem, EMD Biosciences Inc., La Jolla, CA, USA, catalogue no. OP107), anti-SIRT1 (Abcam Ltd., Cambridge, UK, catalogue no. ab32441), and HRP-conjugated anti-Flag (Abcam Ltd., catalogue no. ab49763). Anti-BRCA1 (catalogue no. sc-642), anti-p53 (catalogue no. sc-126), and anti-Actin (catalogue no. sc-47778) were purchased from Santa Cruz Biotechnology Inc. (Santa Cruz, CA, USA). Alexa Fluor 488 conjugated donkey anti-mouse IgG (catalogue no. A-21202) and Alexa Fluor 568 conjugated goat anti-rabbit IgG (catalogue no. A-11011) were purchased from Invitrogen (Carlsbad, CA, USA).

### Immunoprecipitation and western blot

The formation of a TFII-I-BRCA1 complex in HeLa, HCC1937, and COS7 cells was analysed by immunoprecipitation. The whole-cell extracts of HeLa and HCC1937 cells were applied for immunoprecipitation with anti-BRCA1 antibodies or preimmune IgG. The immunoprecipitates were subjected to 30 *μ*l of protein G sepharose 4 Fast Flow (GE healthcare UK Ltd., Buckinghamshire, UK) and subsequently immunoblotted by anti-TFII-I antibodies. Reciprocal immunoprecipitation was also performed. COS7 cells transfected with indicated plasmids were lysed, fractionated, and subjected to anti-FLAG M2 agarose (Sigma-Aldrich, St Louis, MO, USA). Immunoprecipitated materials were blotted with anti-GST antibodies to identify TFII-I-containing complexes.

### RNAi

The ablation of TFII-I and BRCA1 was performed by transfection of HeLa cells with siRNA duplex oligos synthesised by Qiagen (Hilden, Germany). Cells were transfected with control siRNA (AllStars Negative Control siRNA, 1027281), TFII-I-specific siRNA (TFII-I-RNAi: 5′-AAACGGAGCCUACUGAACA-3′, which covered mRNA regions of nucleotides 956–974 (amino acids 195–201) of TFII-I), DBC1-specific siRNA (DBC1-RNAi: 5′-AAACGGAGCCUACUGAACA-3′, which covered mRNA regions of nucleotides 1379–1397 (amino acids 460–466) of DBC1), and BRCA1-specific siRNA (SI02664361) using HiperFect reagent (Qiagen).

### GST-pull down assay

Glutathione-S-transferase fusion proteins or GST alone were expressed in *Escherichia coli* and immobilised on glutathione-sepharose 4B beads (GE healthcare UK Ltd.). The GST proteins were incubated with nuclear extracts of HeLa cells. Unbound proteins were removed and specifically bound proteins were eluted and analysed by SDS–PAGE.

### Luciferase assay

Transfection was performed with Effectene reagent (Qiagen) or Lipofectamine 2000 (Invitrogen) according to the manufacturer's recommendation. For luciferase assay, cells were transfected with indicated expression vectors and/or GAL4 vectors. Reporter plasmids (17M8-AdMLP-luc or SIRT1-luc) were also cotransfected. As an internal control to equalise transfection efficiency, phRL CMV-*Renilla* vector (Promega Co., Madison, WI, USA) was also transfected in all the experiments. Individual transfections, each consisting of triplicate wells, were repeated at least three times as described previously (Wada *et al*, 2004).

### Fluorescence microscopy

HeLa cells were grown on 12 mm BD BioCoat glass coverslips (BD Biosciences, San Jose, CA, USA, 354085) in 6-well plates. The cells were exposed to 8-gray (Gy) of *γ*-irradiation, fixed with PBS containing 4% paraformaldehyde and permeabilised in PBS with 0.2% (v/v) Triton X-100. After blocking, the cells were incubated sequentially with anti-BRCA1 and anti-TFII-I antibodies. Secondary antibodies were Alexa Fluor 488 conjugated donkey anti-mouse IgG, or Alexa Fluor 568 conjugated goat anti-rabbit IgG. The slides were briefly counter-stained and analysed under the confocal fluorescence microscope (Carl-Zeiss MicroImaging Inc., Oberkochen, Germany). Quantification of colocalisation was analysed using LSM7 series-ZEN200x software (Carl-Zeiss MicroImaging Inc.), and the ratio of colocalisation pixels *vs* total pixels in the target area was measured.

### Chromatin immunoprecipitation assay

Preparation of soluble HeLa chromatin for PCR amplification was performed essentially as described (Oishi *et al*, 2006; Hiraike *et al*, 2010). Subconfluent HeLa cells were cross-linked with 1.5% formaldehyde at room temperature for 15 min, and washed twice with ice-cold PBS. The cell pellet was then resuspended in 0.2 ml lysis buffer and sonicated by Bioruptor UCD-250 (Cosmo Bio Co. Ltd., Tokyo, Japan). The sheared soluble chromatin was then subjected to immunoprecipitation with specific antibodies and protein G-sepharose equilibrated with salmon sperm DNA (Millipore, Upstate, Billerica, MA, USA). After extensive wash, the beads were eluted. The eluate was incubated for 6 h at 65°C to reverse the formaldehyde cross-linking. The extracted DNA was purified with the use of QIAquick PCR purification kit (Qiagen). Polymerase chain reaction was performed using specific primers for the SIRT1 promoter (Wang *et al*, 2008; Hiraike *et al*, 2010) and the p21 promoter (Oishi *et al*, 2006).

## Results

### TFII-I and BRCA1 interact *in vivo*

**Figure 1:**
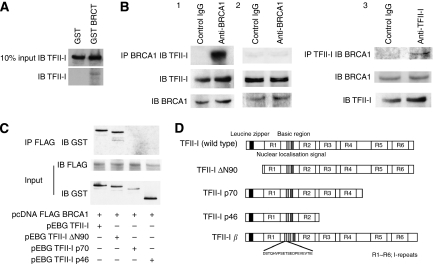
*In vivo* and *in vitro* association between TFII-I and BRCA1, and mapping of the BRCT-interacting region of TFII-I. (**A**) Identification of the interaction between BRCT and TFII-I using GST-BRCT. Bacterially expressed GST fusion proteins immobilised on beads were used in *in vitro* pull-down assays. Nuclear extracts of HeLa cells were incubated with GST-BRCT. The beads were extensively washed, and followed by immunoblotting (IB) using anti-TFII-I antibodies. (**B**) The complex formation of TFII-I and BRCA1 in HeLa cells was analysed by co-immunoprecipitation (IP) with the antibodies to BRCA1 (epitope mapping at the carboxyl-terminus of BRCA1). The immunoblotting analysis using anti-TFII-I antibodies revealed the existence of TFII-I in cell lysate immunoprecipitates (Figure 1B, 1), which indicates that TFII-I physically associates with BRCA1 in living cells. Reciprocal immunoprecipitation analysis confirmed the association of TFII-I and BRCA1 (Figure 1B, 3). The whole-cell extracts of HCC1937 cells known to lack last BRCT domain were also immunoprecipitated with anti-BRCA1 antibodies (epitope mapping at the amino-terminus of BRCA1). The immunoblotting analysis revealed the absence of TFII-I in cell lysate immunoprecipitates (Figure 1B, 2), indicating the importance of BRCT as a binding surface of TFII-I. (**C**) Mapping of the BRCT-interaction region of TFII-I. COS7 cells were transfected with GST-tagged TFII-I (wild type, ΔN90, p70, and p46) and Flag-tagged BRCA1 expression vectors. Nuclear extracts of transfected COS7 cells were prepared and the complex formation of TFII-I and BRCA1 was analysed by IP with the anti-Flag M2 agarose beads, followed by IB using anti-GST antibodies. (**D**) A schematic diagram of the structure of TFII-I (wild type, ΔN90, p70, p46, and *β* isoform) is shown.

**Figure 2:**
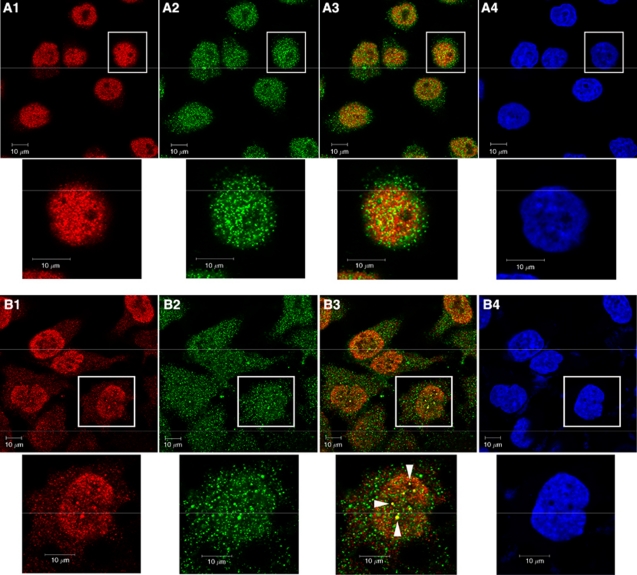
Colocalisation of BRCA1 and TFII-I in HeLa cells. (**A**–**D**) HeLa cells were either treated by 8-Gy of gamma-irradiation or none, fixed, and permeabilised. The cells were incubated with primary antibodies and subsequently with secondary antibodies. The expression of BRCA1 (green) and TFII-I (red) was investigated under the confocal fluorescence microscopy (Carl-Zeiss). Representative immunofluorescence studies are shown (**A**, control cells; **B**, irradiated cells; 1, TFII-I; 2, BRCA1; 3, merge; 4, 4′, 6-diamino-2-phenylindole staining). Arrows in B3 indicate a cell showing nuclear foci formation of TFII-I and BRCA1. Bars indicate 10 *μ*m.

**Figure 3:**
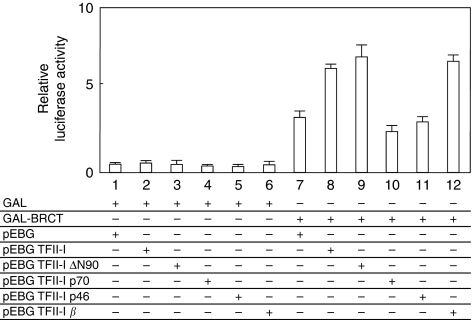
TFII-I stimulates transcription of GAL4-BRCT through its carboxyl-terminal domain. Transient transfection assays were performed to examine the cofactor activity of TFII-I in the transactivation function of GAL4-fused BRCT. COS7 cells were transfected with the indicated combinations of mammalian expression plasmids. At 24 h after transfection, the cells were harvested, and transfected whole-cell lysates were assayed for luciferase activity produced from the reporter plasmid (17M8-AdMLP-luc). TFII-I showed a specific stimulation of the transactivation function of BRCT. Carboxyl-terminus of TFII-I was indispensable for this stimulation of BRCT. The phRL *Renilla* CMV-luc vector was transfected as a control of transfection efficiency. Each experiment was repeated at least three times in triplicate. Error bars represent s.d.

The pull-down products of nuclear extracts from HeLa cells through GST-BRCT column were separated by SDS–PAGE. In consistent with our preliminary result, we confirmed that TFII-I proteins interact with GST-BRCT by western blot (Figure 1A). To determine the interaction between the endogenous TFII-I and BRCA1 in cultured human cells, whole-cell lysates of HeLa cells were immunoprecipitated with anti-BRCA1 antibodies (epitope mapping at the carboxyl-terminus of BRCA1). The immunoblotting analysis using anti-TFII-I antibodies revealed the existence of TFII-I in cell lysate immunoprecipitates (Figure 1B, 1), which indicates that TFII-I physically associates with BRCA1 in living cells. The whole-cell extracts of HCC1937 cells known to lack last BRCT domain were also immunoprecipitated with anti-BRCA1 antibodies (epitope mapping at the amino-terminus of BRCA1). The immunoblotting analysis revealed the absence of TFII-I in cell lysate immunoprecipitates, which reinforced the importance of BRCT domain as the interaction surface with TFII-I (Figure 1B, 2). These data indicated that TFII-I interacted with BRCA1 through the BRCT domain. Reciprocal immunoprecipitation analysis confirmed this association (Figure 1B, 3).

To identify the regions of TFII-I that are responsible for the interaction with BRCT, deletion mutants of TFII-I were used for the assay. COS7 cells were transfected with Flag-tagged BRCA1 and GST-tagged TFII-I and nuclear extracts of transfected cells were immunoprecipitated with the anti-FLAG M2 agarose beads. Western blotting analysis with anti-GST antibodies revealed the existence of GST-tagged TFII-I in the protein extract of immunoprecipitates (Figure 1C), confirming that BRCA1 forms a complex with TFII-I. Flag-tagged BRCA1 and deletion mutants of GST-tagged TFII-I were also subjected to immunoprecipitation. TFII-I lacking the amino terminal region (TFII-I ΔN90) also interacted with BRCA1, while TFII-I lacking carboxyl terminus (TFII-I p70 and p46) showed no interaction with BRCA1. Immunoprecipitation using cytoplasmic fraction of the COS7 cells was also performed and TFII-I was not co-immunoprecipitated with BRCA1. These findings indicate that the carboxyl terminus (R3-R6) of TFII-I and the BRCT domain are both necessary and sufficient for the interaction between TFII-I and BRCA1 in cultured cells.

### TFII-I and BRCA1 form endogenous complex in intact cells and nuclear foci in DNA-damaged cells

As BRCA1 and TFII-I interact *in vivo*, it was of interest to examine the subcellular distribution of these two proteins. To this end, immunofluorescence analysis was performed on HeLa cells. More than 80% substantial colocalisation signal (Merge) of BRCA1 (Alexa Fluor 488-conjugated anti-mouse IgG, green) and TFII-I (Alexa Fluor 568-conjugated anti-rabbit IgG, red) was observed in nuclei in control cells. Therefore, protein complexes containing both BRCA1 and TFII-I are likely to be distributed throughout nuclei. This result also implied the importance of nuclear localisation signal for the colocalisation of BRCA1 and TFII-I in nuclei. The previous study demonstrated that BRCA1 has been shown to display discrete nuclear foci after treatment of cells with irradiation (Zhong *et al*, 1999). HeLa cells irradiated with 8-Gy *γ* radiation demonstrated the punctate pattern of immunostaining for BRCA1 and this pattern overlap TFII-I-containing foci in HeLa cells (Figure 2B, 1–3). These data indicated that double strand DNA damage lead to the nuclear accumulation of the BRCA1-TFII-I complex, possibly at the site of double strand DNA-damage.

### TFII-I enhances the transcriptional activation of BRCT

The result that the BRCT domain interacts with TFII-I led us to examine role of TFII-I in the transactivation function of GAL4-fused BRCT. Transient transfection assays were performed using a 17M8-AdMLP-luc luciferase reporter plasmid, carrying eight tandem repeat GAL4 DNA-binding sites (17M × 8) upstream of AdMLP driving expression of the firefly luciferase gene. The TFII-I alone had no effect on this luciferase reporter construct (Figure 3, lanes 2–6). Although GAL4-BRCT fusion protein (GAL-BRCT) activated the promoter activity of the reporter plasmid in COS7 cells, the transcriptional activity of BRCT was significantly stimulated by the expression of TFII-I at best two-fold on the artificial promoter in luciferase assays (Figure 3, lanes 7–8). Although TFII-I ΔN90 possessing the BRCT binding domain stimulated the transcriptional activation of GAL-BRCT, TFII-I lacking an interaction domain with BRCA1 (TFII-I p70 and p46) lost its ability to stimulate the BRCT-mediated transcriptional activation (Figure 3, lanes 9–11). The mammalian TFII-I has four spliced isoforms: *α*, *β*, *γ*, and Δ (wild type). Upon serum starvation, the *β* and Δ isoforms exhibits subcellular distribution changes in murine NIH3T3 cells (Hakre *et al*, 2006). The experimental data showed that TFII-I *β* also stimulated the transcriptional activation of BRCT (Figure 3, lane 12). These results suggest that carboxyl terminus of TFII-I has a significant role in the stimulation of BRCT-dependent transactivation.

### TFII-I enhances BRCA1-mediated SIRT1 expression

**Figure 4:**
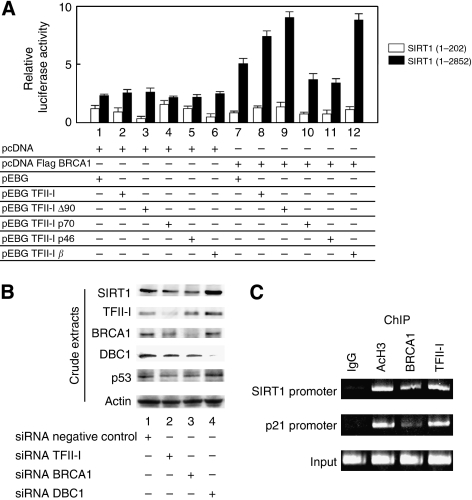
TFII-I stimulates transcription by BRCA1 through its carboxyl-terminal domain. (**A**) Transient transfection assays were performed to examine the influence of TFII-I using an artificial luciferase reporter constructs. COS7 cells were transfected with the indicated combinations of mammalian expression plasmids. At 24 h after transfection, cells were harvested, and transfected whole-cell lysates were assayed for luciferase activity produced from the reporter plasmids. Full-length TFII-I and TFII-I ΔN90 showed specific upregulation of SIRT1 (1-2852)-luciferase activity mediated by BRCA1, while TFII-I p70 and TFII-I p46, lacking BRCT-interaction region, had no effect on SIRT1-luciferase activity. TFII-I showed no effect on SIRT1 (1-202)-luciferase activity that lack the binding domain of BRCA1. (**B**) The siRNA-mediated knockdown of BRCA1 decreased the expression of SIRT1. Knockdown of TFII-I also resulted in downregulation of SIRT1. Expression of BRCA1 and p53 was decreased by depletion of TFII-I. HeLa cells were transfected with indicated siRNA. At 48 h after transfection, cells were harvested and analysed by western blotting. (**C**) Chromatin immunoprecipitation assay was performed to confirm the recruitment of BRCA1 and TFII-I at the SIRT1 gene promoter and the p21 gene promoter. Both promoter regions are known to recruit BRCA1.

The previous chromatin immunoprecipitation assay showed that BRCA1 interacted with the SIRT1 promoter region between 1354 and 1902 and this binding resulted in elevated expression of SIRT1 (Wang *et al*, 2008; Hiraike *et al*, 2010). We explored whether TFII-I has an effect on the BRCA1-mediated stimulation of the SIRT1 promoter and demonstrated that the BRCA1-mediated stimulation of the SIRT1 promoter was specifically upregulated by exogenous expression of TFII-I although the impact of TFII-I was rather less on SIRT1 promoter compared with AdMLP (Figure 4A, lane 8). The TFII-I p70 and p46 showed no influence to enhance the BRCA1-mediated transactivation of SIRT1-luciferase reporter constructs, but TFII-I ΔN90 showed enhancement on the SIRT1-luciferase transactivation function mediated by BRCA1 (Figure 4, lanes 9–11). The TFII-I *β* was also able to stimulate the SIRT1-luciferase transcriptional activation function mediated by BRCA1 (Figure 4, lane 12). Interaction of BRCA1 with the SIRT1 promoter region was postulated to be important for the activation function of TFII-I because SIRT1-luciferase (1–202) reporter constructs lacking the BRCA1-interaction site showed no enhancement of luciferase activity.

We next examined the effect of siRNA-mediated depletion of TFII-I or BRCA1 on their downstream genes. Knockdown of BRCA1 expression by BRCA1-specific siRNA abrogated the expression of SIRT1 (Figure 4B, lane 3), validating the previous report that the expression of SIRT1 is indeed dependent on BRCA1 (Wang *et al*, 2008). As shown in Figure 4B, lane 2, depletion of the endogenous TFII-I also decreased the expression of SIRT1. Thus, our data demonstrate that TFII-I has a critical role in regulating downstream gene expressions dependent on BRCA1 *in vivo*. Depletion of TFII-I resulted in downregulation of p53 and BRCA1, suggesting a role of TFII-I in DNA damage response (Figure 4B, lane 2).

To test whether TFII-I and BRCA1 were indeed recruited to the SIRT1 promoter, we performed a chromatin immunoprecipitation assay using the SIRT1 gene promoter 1354–1902, a region known to recruit BRCA1 (Wang *et al*, 2008; Hiraike *et al*, 2010) and the p21 gene promoter (Oishi *et al*, 2006). As expected, clear recruitment of endogenous TFII-I and BRCA1 to the target sequence (1354–1902) in the SIRT1 and the p21 promoter was observed in HeLa cells (Figure 4C).

## Discussion

The TFII-I is considered to have important roles in regulating the expression of genes as a signal-induced multifunctional transcription factor (Hakre *et al*, 2006; Roy, 2007). Here, we demonstrated that endogenous TFII-I associated with BRCA1, which suggests the possibility that TFII-I has a functional relationship with BRCA1-related phenotypical changes. This interaction between BRCA1 and TFII-I was physiologically functional because our results demonstrated that the TFII-I-containing complex stimulated the autonomous transcriptional activity of BRCT.

The transcriptional activation function of BRCT is considered to be a key to its tumour suppressor activity (Chapman and Verma, 1996; Monteiro *et al*, 1996) because a point mutation within the BRCT domain (A1708E), lacking transactivation function, was shown to be critical for a DNA damage response by the treatment with methylmethane sulfonate (Zhong *et al*, 1999). The importance of BRCT for transcriptional control and growth suppression is also highlighted by the fact that cancer associated mutations attenuated both, but neutral polymorphism did not (Humphrey *et al*, 1997). The BRCT domain is found in a diverse group of proteins implicated in DNA repair and cell cycle check-point control (Bork *et al*, 1997; Callebaut and Mornon, 1997). BRCA1 carboxyl-terminal domain possesses an autonomous folding unit defined by conserved clusters of hydrophobic amino acids, and BRCT is likely to represent a protein interaction surface (Saka *et al*, 1997). Thus, activation of BRCT has implications both in anti-tumorigenic and in DNA repair processes. Anti-tumorigenic role of TFII-I is also supported by the finding that TFII-I inhibits the growth of MCF-7 (Ogura *et al*, 2006). The study had shown that TFII-I downregulates a subset of oestrogen-responsive genes, only those containing initiator elements, by recruiting oestrogen receptor *α* and co-repressors to these promoters. Therefore, TFII-I is not thought to be a general activator of transcription and the transcriptional control by TFII-I would be promoter-dependent manner. The transcriptional regulation played by TFII-I may be complicated because TFII-I was shown to co-immunoprecipitates with transcriptional repressor, histone deacetylase 3 (Wen *et al*, 2003).

We also have shown that TFII-I and BRCA1 colocalise in nuclei of irradiated cells. Previous studies indicated that BRCA1 responds to the repair of DNA by homologous recombination. The BRCA1 associates with RAD51 in subnuclear clusters (Zhong *et al*, 1999). The RAD51 is postulated to be a key component of the mechanism in which DNA damage is repaired by homologous recombination. When cells are exposed to ionising radiation, both BRCA1 and RAD51 localise to the damaged region, and both BRCA1 and RAD51 initiate homologous recombination and the repair of double-strand breaks. Our data that TFII-I and BRCA1 formed nuclear foci after irradiation treatment may suggest that both BRCA1 and TFII-I participates in the DNA damage repair pathway. These data are consistent with the previous observation that TFII-I influences persistence of *γ*-H2AX foci and thus affects double strand break repair, suggesting a role for TFII-I in DNA repair (Desgranges and Roy, 2006). Our results also indicated that TFII-I promoted BRCA1-dependent transcriptional regulation because SIRT1-luciferase activity was potentiated by the ectopic expression of TFII-I. The TFII-I would serve as a transcriptional activation factor to manipulate transcriptions, thereby influencing transcriptional products such as SIRT1. These results also suggest the possible mechanism how TFII-I regulates DNA damage machinery because SIRT1 possesses DNA repair activity (Jeong *et al*, 2007). In consistent with these data, depletion of endogenous TFII-I resulted in a decreased expression of p53 and BRCA1. We have to further confirm the effect of DNA damage response when TFII-I is abrogated to show its involvement in this mechanism. Our results showed a new insight that TFII-I may serve, at least in part, as DNA damage response machinery.

In conclusion, our data indicate that TFII-I have an important role in regulating BRCA1-mediated functions through binding to the BRCT domain and it may be probable that TFII-I is involved in anti-tumorigenic processes. The meaning of TFII-I functions would be apparent by evaluating its expression in tumour tissues such as breast cancer. The expression of TFII-I would be therapeutically beneficial by affecting different TFII-I-mediated regulatory pathways together with BRCA1 and the failure of binding between BRCA1 and TFII-I may be a key event in cancer predisposition.
